# The embodiment of the neighborhood socioeconomic environment in the architecture of the immune system

**DOI:** 10.1093/pnasnexus/pgae253

**Published:** 2024-06-27

**Authors:** Grace A Noppert, Philippa Clarke, Rebecca C Stebbins, Kate A Duchowny, Robert Melendez, Kimberly Rollings, Allison E Aiello

**Affiliations:** Survey Research Center, Institute for Social Research, University of Michigan, 426 Thompson St., Ann Arbor, MI 48104, USA; Survey Research Center, Institute for Social Research, University of Michigan, 426 Thompson St., Ann Arbor, MI 48104, USA; Robert N. Butler Columbia Aging Center, Mailman School of Public Health, Columbia University Irving Medical Center, 722 W. 168th St., New York, NY 10032, USA; Survey Research Center, Institute for Social Research, University of Michigan, 426 Thompson St., Ann Arbor, MI 48104, USA; Survey Research Center, Institute for Social Research, University of Michigan, 426 Thompson St., Ann Arbor, MI 48104, USA; Survey Research Center, Institute for Social Research, University of Michigan, 426 Thompson St., Ann Arbor, MI 48104, USA; Robert N. Butler Columbia Aging Center, Mailman School of Public Health, Columbia University Irving Medical Center, 722 W. 168th St., New York, NY 10032, USA

## Abstract

There is growing recognition of the importance of immune health for understanding the origins of ageing-related disease and decline. Numerous studies have demonstrated consistent associations between the social determinants of health and immunosenescence (i.e. ageing of the immune system). Yet few studies have interrogated the relationship between neighborhood socioeconomic status (nSES) and biologically specific measures of immunosenescence. We used data from the US Health and Retirement Study to measure immunosenescence linked with neighborhood socioeconomic data from the National Neighborhood Data Archive to examine associations between indicators of nSES and immunosenescence. We found associations between both the ratio of terminally differentiated effector memory to naïve (EMRA:Naïve) CD4+ T cells and cytomegalovirus (CMV) immunoglobulin G (IgG) levels and nSES. For the CD4+ EMRA:Naïve ratio, each 1% increase in the neighborhood disadvantage index was associated with a 0.005 standard deviation higher value of the EMRA:Naïve ratio (95% CI: 0.0003, 0.01) indicating that living in a neighborhood that is 10% higher in disadvantage is associated with a 0.05 higher standardized value of the CD4+ EMRA:Naïve ratio. The results were fully attenuated when adjusting for both individual-level SES and race/ethnicity. For CMV IgG antibodies, a 1% increase in neighborhood disadvantage was associated a 0.03 standard deviation higher value of CMV IgG antibodies (*β* = 0.03; 95% CI: 0.002, 0.03) indicating that living in a neighborhood that is 10% higher in disadvantage is associated with a 0.3 higher standardized value of CMV. This association was attenuated though still statistically significant when controlling for individual-level SES and race/ethnicity. The findings from this study provide compelling initial evidence that large, nonspecific social exposures, such as neighborhood socioeconomic conditions, can become embodied in cellular processes of immune ageing.

**Significance Statement**

Understanding the life course drivers of ageing-related disease and decline is of critical importance for population health. The immune system is a particularly relevant system for understanding these processes as immune system ageing is both a risk factor for a number of ageing-related diseases and is highly sensitivity to the social environment. In the current study, we were able to discern consistent associations between a nonspecific social exposure, neighborhood socioeconomic status, and biologically specific processes of immune ageing. This result has implications for how we understand the drivers of immune ageing across the life course. Neighborhood environments, in contrast to other social determinants of health, are potentially modifiable exposures, and our results could be used to identify and address neighborhoods that may make residents particularly vulnerable to poor immune health leading to health inequities across the life course.

## Introduction

Biomarkers of immune ageing, or immunosenescence, are a growing and promising area of inquiry in population health research. The changes observed in the immune system as individuals age have been linked to a number of ageing-related diseases and age-related decline more generally, including cardiovascular diseases, cancers, type 2 diabetes mellitus, neurodegenerative diseases, frailty, and premature mortality (1, 2). The “inflammaging” hypothesis was coined to describe the chronic, low-grade inflammation that individuals experience as they age and which many studies have found to be a risk factor for a number of other ageing-related conditions (3). Moreover, a growing number of studies have demonstrated strong and consistent associations between the social determinants of health (SDOH) at multiple levels (e.g. individual, neighborhood) and indicators of immunosenescence, suggesting that understanding how the social environment impacts immune system ageing may yield insights into how to disrupt long-standing inequities in ageing-related decline and disease.

The neighborhood environment has long been recognized as an important source of both risk and resilience for individual health over the life course (4, 5). Healthy people 2030 names neighborhood environments as a key component of SDOH recognizing that they structure opportunities for employment, health-related behaviors and lifestyles, exposures to environmental toxins, and levels of material and social support (6, 7). A substantial literature documents associations between the neighborhood built and social environments and health outcomes including cardiovascular disease (8), disability (9), and mortality (10). The neighborhood environment can refer to myriad characteristics within a particular geographic context encompassing both material resources and social characteristics of one's local surroundings (11). However, the vast majority of research on neighborhood effects focuses on neighborhood socioeconomic structure, specifically socioeconomic disadvantage, deprivation, or vulnerability, hereafter called neighborhood socioeconomic status (nSES). nSES shapes health not only through the amenities and resources that shape behaviors but also by social processes that result from the concentration of disadvantaged residents in local space (12). Over and above the effects of individual SES, nSES (typically measured as a composite index based on poverty, income, employment, and family structure in the neighborhood) has been repeatedly shown to impact a host of health outcomes including low birth weight, cardiovascular disease, cognitive function, depression, hospital readmission rates, and premature mortality (13).

Interrogating how the neighborhood environment may shape immunosenescence is a growing area of research. The neighborhood environment is a key determinant of both material resource availability, as well as, exposure to environmental toxins (4). Moreover, numerous studies have demonstrated that living in a disadvantaged neighborhood can result in disproportionate stress for its residents (14, 15). All of these neighborhood-level risk factors can impact immune health through various mechanisms making it a promising area for further research. However, to date, the studies that have examined associations between the neighborhood environment and immune health have predominately focused on singular markers of inflammation. While inflammation is a key a component of the immune response, it is a generalized immune response that lacks biological specificity and the direction of the inflammatory response is difficult to ascertain. For example, CRP and IL-6, two widely studied inflammatory markers can be both proinflammatory and anti-inflammatory which can contradict the hypothesized biological mechanisms for many health outcomes (16). With the increasing number of epidemiological studies that include a diverse set of immune biomarkers, it is now possible to interrogate more biologically specific cellular immune markers that may tell us much more about the intricate ways in which the immune system changes with age and in response to the physical and social environment (17, 18).

Phenotypes of T cells that give an indication of the relative age of different T cells are another emerging indicator of immune system ageing that are more biologically specific. Typically, as individuals age there are cellular changes that occur within different components of the immune system, such as T cells, that are relevant for understanding ageing-related decline and disease. Specifically, there tends to be an accumulation of CD8+ T cells over CD4+ T cells, a decrease in the output and number of naïve T cells and an accumulation of terminally differentiated T cells with limited function (19, 20). These changes to the T cell compartment are consistent with a more aged immune phenotype that includes fewer “young” naïve T cells—cells that can better adapt to new infections—and an increase in late-stage antigen-specific CD8+ T cells that have limited functional capacity (1, 21). Chronic herpesvirus infections, and cytomegalovirus (CMV) in particular, are thought to be one of the key drivers of the expansion of antigen-specific T cells. The age-related immune alterations described above all coalesce to form a phenotype of immunosenescence that is associated with multiple other age-related declines and disease (21–23).

Recent studies have begun to explore how the cellular changes occurring in the immune compartment correlate to aspects of the social environment. One recent study, for example, found that racialized minority populations and those of lower SES have more aged immune profiles (e.g. higher CD8+:CD4+ ratio, higher ratios of effector memory (i.e. “older”):naïve (i.e. “younger”) CD4+ and CD8+ ratios) compared to Non-Hispanic White (NHW) and higher SES populations (18). A 2016 study of individuals in Detroit, Michigan found that individuals who experienced post-traumatic stress disorder (PTSD) in the past year showed a shift in the distribution of T cells favoring CD8+ cells over CD4+ cells, and effector memory CD8+ cells over naïve CD8+ cells. These findings suggest that experiencing PTSD was associated with increased immunosenescence compared to those that did not experience PTSD (24). Another study using the Detroit Neighborhood Health Study (DNHS) found that a greater percentage of abandoned homes in a neighborhood, an indicator of nSES, was associated with decreased thymic function, a measure of the output of naïve T cells, consistent with what would be expected with accelerated immune ageing (25).

One critical consideration that emerges in the neighborhood and health literature is the potential for heterogenous effects of the neighborhood environment across demographic characteristics namely age, gender, and racial/ethnic identity (26). While multiple studies have found evidence of heterogeneity in the effects of neighborhood disadvantage by age, much of this literature has focused on younger age cohorts (26, 27) with few studies examining age heterogeneity in cohorts of older adults. Moreover, studies have reported inconsistent findings with regards to heterogeneity in neighborhood effects by race/ethnicity and gender (11). Due to historical processes of racial residential segregation and redlining (28, 29), it is difficult to identify heterogeneity in the effects of neighborhood disadvantage by race/ethnicity because the neighborhoods in which racialized minority populations live in overlap very little with those occupied by white populations (30, 31). Yet again many of these studies have not examined these relationships in older populations. In the ageing literature, there have been a few studies that reported heterogenous effects of the neighborhood environment by gender. Several of these studies report that women seem to benefit more than men from living in higher SES neighborhoods (32–34). Taken together, however, there are no consistent findings with regards to heterogenous effects of the neighborhood environment by age, gender, and race/ethnicity in older populations highlighting the need for further study in this area.

While the literature on the relationship between the neighborhood environment and immunosenescence is scant, there are a few studies, including those cited above, that suggest that the neighborhood does indeed interact with biological processes associated with accelerated immune ageing, likely through the chronic activation of the stress response and the accompanying physiological dysregulation that occurs across a number of body systems (25, 35, 36). However, many of the previous studies were conducted in small, nonrepresentative samples, limiting their generalizability. Thus, to further interrogate these relationships, we conducted an analysis examining whether neighborhood SES is associated with patterns of immunosenescence in a national sample of older adults in the United States. We used venous blood sample data from the Health and Retirement Study (HRS) linked with objective measures of nSES from the National Neighborhood Data Archive (NaNDA) to address the following research questions (i) are measures of neighborhood SES associated with biomarkers of immunosenescence in a sample of older adults in the United States, net of the potential confounders of individual-level SES and race/ethnicity? (ii) Do these associations differ by age group, sex, and race/ethnicity?

Of note is our decision to model race/ethnicity as a potential confounder of the association between neighborhood-level SES and immunosenescence in our main statistical models. Racial/ethnic categorizations are the result of the social process of racialization that has systematically placed certain individuals in separate and unequal contexts resulting in disproportionate exposures to social, environmental, and economic exposures (37). In the current study, we conceptualize race/ethnicity status as a proxy, though imperfect, for the experience of structural racism which systematically shapes which neighborhoods individuals can live in. Simultaneously, the stress of structural racism has real consequences for the immune health of racialized minority populations (38). In response to the second research question, we also explore race/ethnicity as a potential effect modifier.

## Materials and methods

### Study sample

The HRS is an on-going, nationally representative longitudinal survey of US adults over the age of 50, which began in 1992 and includes over 20,000 adults. Follow-up occurs every 2 years. We used data from the subsample of HRS participants who participated in the Venous Blood Study (VBS), which assessed biomarkers of immune function in 2016. Demographic data were drawn from the 2016 HRS tracker file and the 2016 RAND longitudinal file. The neighborhood social environment was measured based on the address at which the participant reported living in 2016 and was geocoded to the census tract (as a proxy for neighborhood). There were 9,933 participants who completed the VBS and had valid test results. Our analytical sample consisted of individuals who had valid outcome measures for each of the four immune measures, as well as complete neighborhood, demographic, and health data. Full details of the sample construction and data collection procedures are provided in Fig. S1.

### Exposures: neighborhood socioeconomic status

We used nSES measures from the NaNDA, a publicly available repository of neighborhood characteristics (https://nanda.isr.umich.edu/). We focused on two primary measures of nSES at the census tract level: neighborhood disadvantage and neighborhood affluence. Details on how each of these measures were derived is included in the Supplementary material.

### Outcomes: biomarkers of immune function

We utilized three measures of immune function: CD8+:CD4+, EMRA CD4+:Naïve CD4+, and EMRA CD8+:Naïve CD8+. Further details on how the measures were derived and from which sample populations can be found in the Supplementary material. All ratio measures were standardized to facilitate interpretation of the coefficients and comparison across measures. For all three immune measures we constructed, higher values correspond to a more aged immune profile.

#### Cytomegalovirus

In addition, we examined CMV IgG antibody levels both as an immune outcome as well as a potential meditator of the relationship between nSES and immunosenescence. The role of CMV in immunosenescence remains unclear. This large DNA virus may drive immunosenescence or interact biologically with the immune system in ways that accelerate immunosenescence. Alternatively, immunosenescence may lead to loss of immune control over CMV and subclinical reactivation. Importantly, CMV has known associations with socioeconomic factors at the population-level (39). CMV immunoglobulin (Ig) G levels were measured in serum using the Roche e411 immunoassay analyzer.

### Sociodemographic covariates

Self-reported sociodemographic characteristics including age, sex, race/ethnicity, household income, and educational attainment were obtained from the HRS tracker file. Educational attainment was categorized as less than a high school diploma, high school diploma, some college, or a college diploma and higher. To capture household resource availability, we used the household income to poverty ratio measure as a continuous variable. Sex was self-reported as either male or female. We used a four-level categorical variable to measure race/ethnicity, composed of non-Hispanic black (NHB), Hispanic, Other race/ethnicity, and non-Hispanic white (NHW).

### Health status and health behavior covariates

We also examined associations adjusting for several health behaviors and health status variables that were derived from the 2016 RAND longitudinal file. These included smoking status (categorized as current smoker, former smoker, and never smoker), change in self-reported health, self-report of a change in overall health status, an index of the number of self-reported, physician-diagnosed chronic conditions, and change in functional limitations.

### Statistical analysis

**Fig. 1.**
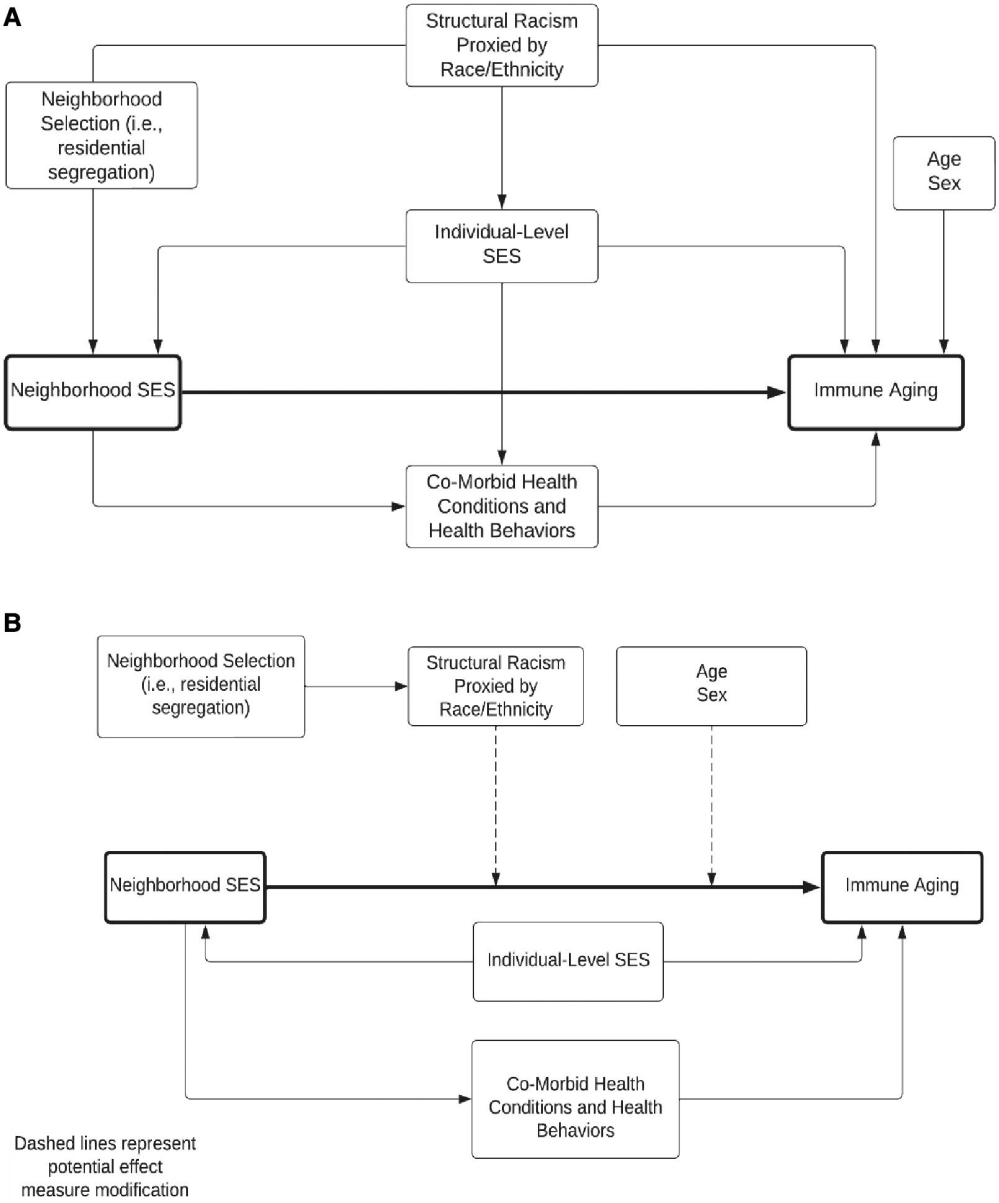
Directed acyclic graph (DAG) depicting the hypothesized relationship between the covariates of interest. A) Corresponds to the DAG for research question 1. B) Corresponds to the DAG for research question 2, the potential for effect measure modification.

Descriptive statistics were calculated for the entire sample and also stratified by the lowest and highest quartiles of neighborhood disadvantage and affluence. The model building strategy, including variable selection, was based on a directed acyclic graph (DAG) analysis. The hypothesized DAG is depicted in (Fig. 1). To estimate the association between neighborhood SES and immune ageing, the minimally sufficient adjustment set includes individual-level SES and race/ethnicity. Figure 1A depicts the DAG for the first research question. Figure 1B depicts the potential effect measure modification of the main association by age, race/ethnicity, and gender.

To examine the first research question, we constructed a series of linear regression models to estimate the associations between neighborhood SES and immune ageing based on the DAG (Fig. 1A). **Model 1** estimates the association between neighborhood SES and immune ageing controlling for age and sex. **Model 2** adds controls for individual-level SES (both education and household income to poverty). **Model 3** adds further controls for individual-level race/ethnicity.

**Model 4** is part of a sensitivity analysis to examine potential mediating pathways by which residence in disadvantaged neighborhoods leads to poor health and worse immune function. **Model 4** includes model 3 with the addition of controls for health behaviors and chronic health conditions. **Model 5** further adds neighborhood population density.

As stated above, we estimated CMV as an immune outcome itself and as a potential mediator in sensitivity analyses. Thus, for the three ratio measures of immune ageing, we also estimated models adjusting for CMV (**model 6**) to assess whether CMV may partially mediate the relationship between neighborhood SES and immunosenescence. CMV is added to the fully controlled model 5.

To address the second research question regarding potential effect measure modification by age, race/ethnicity, and sex, we estimated models with interaction terms for both of the indicators of nSES (Fig. 1B). All of the models evaluating interaction terms were adjusted for age and sex. To assess effect measure modification, we examined the *P*-value for the interaction term.

All analyzes used the 2016 VBS survey weights and were conducted in Stata/MP 17.0 for Windows (64-bit x86-64). A complete case analysis was conducted for each of the four immune outcomes given that the proportion of missingness for each outcome did not exceed 3%. All analyzes were completed in a secure data enclave through the University of Michigan, Institute for Social Research. This study was approved by the University of Michigan Institutional Review Board.

## Results

### Descriptive statistics of the sample population

The sample of 6,176 individuals had a median age of 67 years; 59% were female and 18% NHB, 15% Hispanic, 3% other race/ethnicity, and 64% NHW (Table 1). In descriptive analyzes, we found that the median value (interquartile range [IQR]) for the CD8+:CD4+ ratio was 3.05 (2.8). Participants living in the lowest quartile of neighborhood affluence (Q1) had a median value of 3.36 (3.29) compared to those in the highest quartile (Q4) of neighborhood affluence with a median value of 2.88 (2.51). A similar gradient was observed with neighborhood disadvantage whereby those living in the most disadvantaged neighborhoods had higher median values of the CD8+:CD4+ ratio. Similar trends were also observed for the EMRA:Naïve ratio in both CD8+ and CD4+ cells.

**Table 1.** Descriptive statistics of the study population.

Measure	Full sample	Disadvantage		Affluence	
		Q1 (lowest)	Q4 (highest)	Q1 (lowest)	Q4 (highest)
Neighborhood characteristics					
Disadvantage, median (IQR)	7.81 (7.5)	3.32 (1.48)	15.89 (5.89)	14.39 (8.22)	3.65 (2.13)
Affluence, median (IQR)	28.37 (20.89)	47.54 (18.58)	17.03 (9.93)	15.7 (6.02)	52.52 (12.58)
Density, median (IQR)	2,108.14 (4,476.75)	1,501.98 (2,935.91)	3,714.72 (6,873.55)	2,823.23 (6,363.19)	2,417.05 (3,411.73)
*Immune measures*					
Ratio measures					
CD8+:CD4+ Ratio, median (IQR)	0.33 (0.31)	0.3 (0.31)	0.35 (0.31)	0.35 (0.31)	0.3 (0.3)
EMRA CD4+:Naïve CD4+, median (IQR)	0.04 (0.12)	0.03 (0.08)	0.06 (0.17)	0.06 (0.16)	0.03 (0.07)
EMRA CD8+:Naïve CD8+, median (IQR)	2.31 (4.88)	2.09 (4.87)	2.33 (4.71)	2.49 (4.99)	1.95 (4.5)
CMV					
CMV, % seropositive	0.72	0.58	0.85	0.84	0.59
CMV IgG continuous antibodies U/mL of blood (among seropositives), median (IQR)	214.2 (578.25)	79.4 (443.2)	343.3 (614.2)	349.3 (611.8)	79 (428.7)
*Demographics*					
Age, median (IQR)	67 (15)	69 (16)	64 (14)	65 (15)	67 (15)
Sex					
Female, *N* (%)	5,867 (0.59)	1,287 (0.58)	1,712 (0.62)	1,693 (0.61)	1,138 (0.56)
Male, *N* (%)	4,066 (0.41)	947 (0.42)	1,041 (0.38)	1,075 (0.39)	883 (0.44)
Race/Ethnicity					
Non-Hispanic black, *N* (%)	1,752 (0.18)	100 (0.04)	1,078 (0.39)	899 (0.32)	158 (0.08)
Hispanic, *N* (%)	1,476 (0.15)	118 (0.05)	751 (0.27)	733 (0.26)	147 (0.07)
Other, *N* (%)	316 (0.03)	73 (0.03)	100 (0.04)	81 (0.03)	83 (0.04)
Non-Hispanic white, *N* (%)	6,382 (0.64)	1,941 (0.87)	822 (0.3)	1,053 (0.38)	1,628 (0.81)
Education					
Less than HS, *N* (%)	1,706 (0.17)	155 (0.07)	849 (0.31)	858 (0.31)	113 (0.06)
HS Grad, *N* (%)	5,104 (0.51)	1,039 (0.47)	1,371 (0.5)	1,461 (0.53)	802 (0.4)
Some college, *N* (%)	725 (0.07)	171 (0.08)	179 (0.07)	175 (0.06)	169 (0.08)
College Grad and above, *N* (%)	2,398 (0.24)	869 (0.39)	354 (0.13)	274 (0.1)	937 (0.46)
Household income to poverty ratio, median (IQR)	3.2 (4.11)	4.94 (5.91)	1.97 (2.6)	2.03 (2.45)	5.7 (6.4)
*Health behaviors*					
Smoking status					
Current smoker, *N* (%)	1,152 (0.12)	158 (0.07)	441 (0.16)	451 (0.16)	156 (0.08)
Former smoker, *N* (%)	4,340 (0.44)	1,005 (0.45)	1,160 (0.42)	1,131 (0.41)	876 (0.43)
Never smoker, *N* (%)	4,390 (0.44)	1,054 (0.47)	1,141 (0.41)	1,175 (0.42)	972 (0.48)
*Health status indicators*					
Change in self-reported health, median (IQR)	0 (0)	0 (0)	0 (0)	0 (0)	0 (0)
Self-report of a change in overall health status, median (IQR)	3 (0)	3 (0)	3 (0)	3 (0)	3 (0)
Chronic conditions index, median (IQR)	2 (2)	2 (2)	3 (2)	3 (2)	2 (2)
Change in functional limitations, median (IQR)	0 (0)	0 (0)	0 (0)	0 (0)	0 (0)

CD8+ CD4+ Ratio, *n* = 9,072. CD4+ EMRA:Naïve, *n* = 9,029. CD8+ EMRA:Naïve, *n* = 9,030. CMV IgG, *n* = 9,589.

### Associations between nSES and the CD8+:CD4+ ratio

**Fig. 2.**
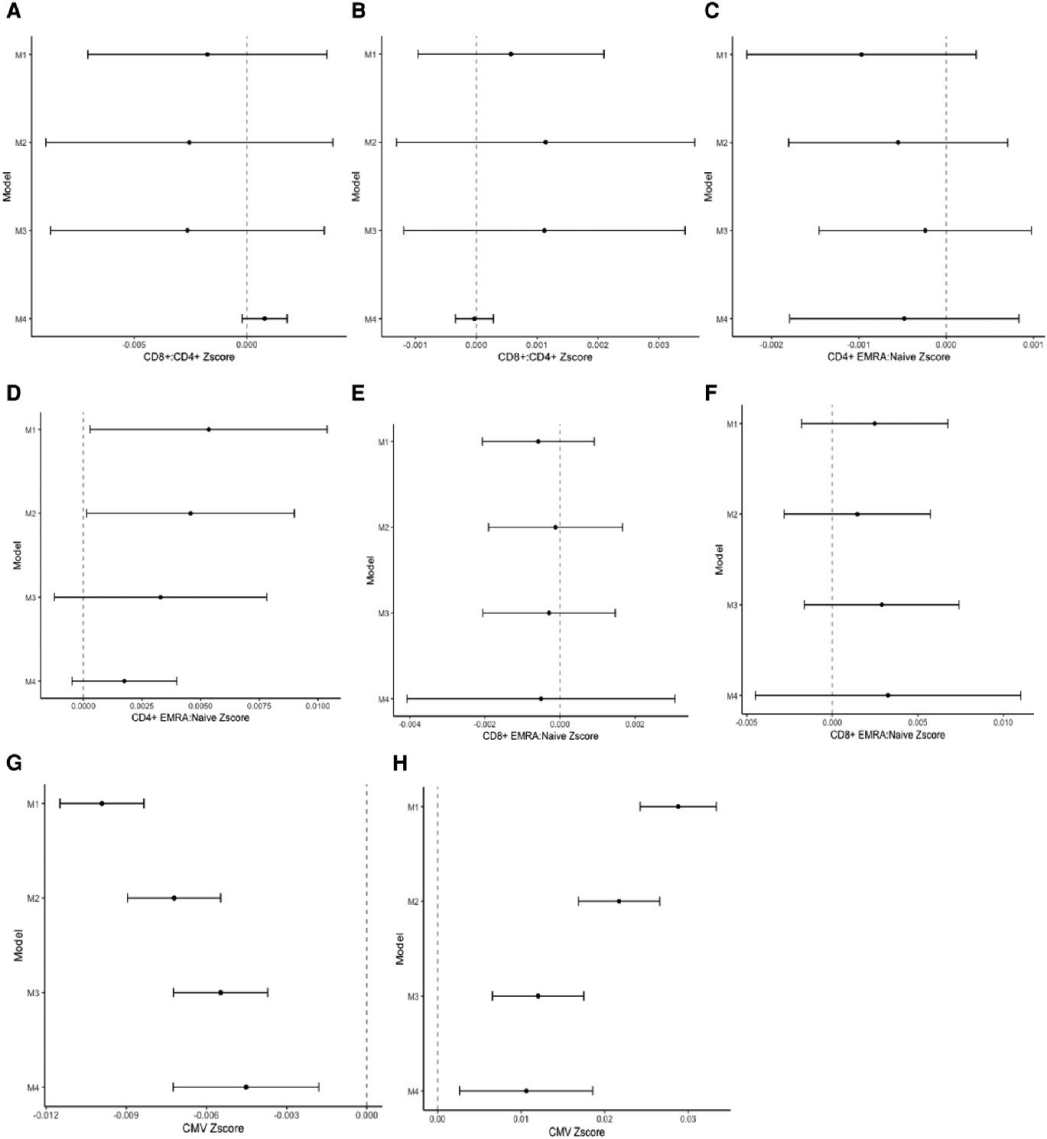
Results of the regression analyzes estimating the association between neighborhood disadvantage and affluence, and each of four immune biomarkers. A) Corresponds to the results estimating the association between neighborhood affluence and CD8+: CD4+. B) Corresponds to the results estimating the association between neighborhood disadvantage and CD8+: CD4+. C) Corresponds to the results estimating the association between neighborhood affluence and CD4+ EMRA:Naïve Ratio. D) Corresponds to the results estimating the association between neighborhood disadvantage and CD4+ EMRA:Naïve Ratio. E) Corresponds to the results estimating the association between neighborhood affluence and CD8+ EMRA:Naïve Ratio. F) Corresponds to the results estimating the association between neighborhood disadvantage and CD8+ EMRA:Naïve Ratio. G) Corresponds to the results estimating the association between neighborhood affluence and CMV. H) Corresponds to the results estimating the association between neighborhood disadvantage and CMV.

Figure 2 depicts the regression coefficients (and 95% CIs) for models examining each measure of nSES and their association with biomarkers of immunosenescence. Coefficients represent the standard deviation change in each biomarker of immunosenescence associated with a one-unit change in the neighborhood socioeconomic indicator. For interpretation purposes, higher values on each of the immune indicators are consistent with a more aged immune profile. There were no statistically significant associations between neighborhood affluence or disadvantage and the CD8+:CD4+ ratio (Fig. 2A and B).

### Associations between nSES and the CD4+ EMRA:naïve ratio

In the CD4+ compartment, we found no statistically significant associations between neighborhood affluence and the EMRA:Naïve ratio (Fig. 2C). However, we did find statistically significant associations with neighborhood disadvantage (Fig. 2D). In model 1 controlling for age and sex, each 1% increase in the neighborhood disadvantage index was associated with a 0.005 standard deviation higher value of the EMRA:Naïve ratio (95% CI: 0.0003, 0.01).

Models 2 and 3 for each panel present the results for the associations between nSES and immunosenescence net of individual-level SES and race/ethnicity. For the CD4+ compartment, the association with neighborhood disadvantage was attenuated though robust in model 2 adjusting for individual-level SES (*β* = 0.005; 95% CI: 0.00001, 0.009) indicating that living in a neighborhood that is 10% higher in disadvantage is associated with a 0.05 higher standardized value of the CD4+ EMRA:Naïve ratio. The effect was fully attenuated in model 3 after adjusting for race/ethnicity (*β* = 0.00329; 95% CI: −0.0001, 0.008) (Fig. 2D).

### Associations between nSES and the CD8+ EMRA:Naïve ratio

In the CD8+ compartment, we observed no statistically significant associations between either neighborhood affluence or disadvantage and the EMRA:Naïve ratio measure (Fig. 2E and F).

### Associations between nSES and CMG IgG

We observed statistically significant associations between both neighborhood affluence and neighborhood disadvantage and CMV IgG response (Fig. 2G and H). In model 1 controlling for age and sex, we found that a 1% increase in neighborhood affluence was associated with a 0.010 standard deviation lower standardized value of CMV IgG antibodies (*β* = −0.01; 95% CI: −0.01, −0.008). A 1% increase in neighborhood disadvantage was associated a 0.03 standard deviation higher value of CMV IgG antibodies (*β* = 0.03; 95% CI: 0.002, 0.03). Put another way, living in a neighborhood that is 10% higher in disadvantage is associated with a 0.3 higher standardized value of CMV IgG.

When examining CMV IgG levels (Fig. 2G and H), associations with neighborhood affluence and disadvantage were attenuated but still statistically significant after adjusting for individual-level SES (model 2) and race/ethnicity (model 3). In model 2, a 1% increase in neighborhood-level affluence was associated with a 0.007 standard deviation lower value of CMV IgG antibodies (*β* = −0.007; 95% CI: −0.009, −0.005), net of individual SES. This was further attenuated in model 3 after adjusting for race/ethnicity (*β* = −0.005; 95% CI: −0.007, −0.004). Similarly, we found that the association with neighborhood disadvantage was attenuated but still statistically significant after adjusting for individual-level SES (model 2) (*β* = 0.02; 95% CI: 0.017, 0.03) and race/ethnicity (model 3) (*β* = 0.01; 95% CI: 0.007, 0.02).

### Sensitivity analyses: associations between nSES and immunosenescence, adjusting for potential mediating variables

In sensitivity analyses, we estimated models adjusting for the potential mediating variables related to health behaviors, chronic health conditions, neighborhood population density, and CMV. In general, we found that the addition of potential mediators did not substantively change the effect estimates. Results are presented in Tables S3–S8, models 4–6.

### Differences in the association between nSES and immunosenescence by age, sex, and race/ethnicity

To address the second study question regarding whether the association between nSES and immunosenescence varies by age, sex, and race/ethnicity, we estimated models that included interaction terms between nSES and age, sex, and race/ethnicity. Of the 40 interactions we tested, only 5 were statistically significant. There were no consistent patterns to these interactions as well. Taken together, there is limited evidence to suggest that the association between nSES and immunosenescence differs by age, sex, and race/ethnicity (see Table S9).

## Discussion

Using data from a national sample of ageing adults in the US HRS, we investigated whether the neighborhood socioeconomic environment was associated with biomarkers of immunosenescence. There were two major findings from this study. First, while we found an association between nSES and immunosenescence, the presence and strength of this association differed according to the specific biomarker investigated. Second, the strongest associations were observed with the CD4+ EMRA:Naïve ratio and CMV and these associations were generally robust to controls for both individual-level SES and race/ethnicity. These findings lay the groundwork for future investigations that can examine the multiple pathways through which the neighborhood environment may become embedded in cellular markers of immunosenescence.

Contrary to our expectation, we did not see any evidence of an association between neighborhood disadvantage and affluence and the CD8+:CD4+ ratio measure. The CD8+:CD4+ ratio (or the inverse CD4+:CD8+) has been used repeatedly in studies of immune ageing (40, 41). However, consistent with previous work, our results suggest that the CD8+:CD4+ ratio may not be as sensitive to the individual- and neighborhood-level socioeconomic environment (42).

We found evidence of more robust associations for the CD4+ EMRA:Naïve ratio than the CD8+ EMRA:Naïve Ratio. CD8+ Cytotoxic T cells induce cell death in virus- or tumor-infected cells (43), and compared to CD4+ T cells, CD8+ T cells age faster—or progress to the terminally differentiated state faster, a process thought to be driven by the reactivation of latent herpesviruses (43, 44). Thus, it may be that among older adults there is less variability among CD8+ T cells, resulting in less robust associations with neighborhood- and individual-levels indicators of SES. In contrast, there may be greater sensitivity among CD4+ T cells to exposure to exogenous stressors and disadvantage in older individuals, because these cells subsets are responsible for multiple functions including activation of aspects of the innate immune system, activation of cytotoxic T cells and B-lymphocytes, and suppression of the immune reaction (45).

Our results align with the growing body of work demonstrating the importance of CMV, and particularly immune control of CMV, for understanding how social processes become embodied. Studies have repeatedly shown that disadvantaged populations including lower SES and racialized minority populations have both higher CMV seropositivity and worse immune control of CMV (39). Recent studies have also found positive associations between poor neighborhood conditions in both childhood and adulthood and CMV seropositivity across the life course (46–48). There are several pathways through which the neighborhood environment may be associated with CMV, and it is likely that these pathways operate across the life course. We hypothesize that the neighborhood environment may both increase the likelihood of exposure to CMV early in the life course and increase the likelihood of repeated infections over the life course (49–51). Given that CMV is a chronic infection that goes through cycles of latency and reactivation over the life course, we also hypothesize that stress of living in a disadvantaged neighborhood could increase the frequency of reactivation resulting in poorer immune control of CMV in older age (24, 52).

Within the growing body of work that examines CMV and its association with both the social environment and immune ageing, there are still many questions unanswered. One critical question is the role CMV plays in the process of immune ageing. Here, we found it most helpful to conceptualize it as an outcome itself given the number of studies that have demonstrated clear associations between CMV IgG and other chronic health conditions (53–55). It may also be that CMV Is a mediator of the relationship between nSES and immune function. Indeed, in the immunology literature there are a number of studies showing that CMV infection drives clonal expansion of T cells resulting in a more aged immune profile, and reduced immune function as individuals age (56–58). Thus, we also did additional analyzes to examine it as a potential mediator. Future work should continue to explore the role of CMV in life course processes linking social disadvantage to immune function.

More broadly, our results suggest that the neighborhood social environment may be associated with subtle changes in aspects of the immune system that may be meaningful for understanding immune health as a whole, as well as potential downstream consequences of immune ageing. While the true clinical and practical significant of these measures is still being uncovered as they are newer measures of immune ageing in the population health literature, one way to think about what these findings mean is in the context of the ageing process. These immune measures emerged from the ageing literature and have often been used to understand immune ageing, and where there might be signs of accelerated immune ageing that are not consistent with what would be expected with age alone. To try and estimate how the magnitude of the associations and compare them to what might be expected with age alone, we ran a series of simple models to estimate how much change we would expect in each of the four immune outcomes with a single year of chronological age (see Table S10). Based on the results above, a 10% higher neighborhood disadvantage score is associated with a 0.05 SD higher CD4+, EMRA:Naïve ratio, or put another way, 6 additional years of chronological age. For CMV, a 10% higher neighborhood disadvantage score is associated with a 0.3 SD higher standardized value of CMV IgG. In other words, living in a neighborhood that is 10% higher in neighborhood disadvantaged is associated with an increased CMV IgG level consistent with about 38 additional years of chronological age. While these are crude associations, it is helpful to contextualize them in the context of changes observed with chronological age.

There are multiple pathways through which the neighborhood socioeconomic and associated physical environment might shape immune ageing. For example, a neighborhood's socioeconomic context can impact an individual's financial and physical access to resources such as healthy food, healthcare, safe places to exercise, and stable housing (4)—factors that improve overall health and wellbeing, including producing a healthier immune system. Disadvantaged neighborhoods also bear a disproportionate burden of environmental hazards many of which are known immune modulators (59). Or perhaps it is the stress of living in disadvantaged neighborhoods—those that lack access to basic resources for health and wellness and those that are burdened with a high a number of environmental hazards—that is causing wear and tear on the immune system (60). Indeed, compelling evidence suggests stress, and particularly exposure to chronic stress, is associated with increases in inflammation as well as immune dysfunction (61, 62). Continuing work is needed that can tease apart the social and biological mechanisms linking the neighborhood environment to immune health. Such studies could also lend insights into how the neighborhood environment could be modified to improve immune health.

We also did not find evidence that the association between nSES and immune health differed by key sociodemographic characteristics: age, sex, and race/ethnicity. However, it may be that we are examining these associations too late in the life course to find heterogeneity in these associations. Studies of the differential impact of neighborhood environments on health by age, sex, and race/ethnicity often report that these differences are most apparent in early life (26). There is a need for more studies that can examine these associations across the life course to understand when, and for whom, the neighborhood environment is exerting the most influence on health.

There are multiple strengths to this study. First, we leverage multiple markers of immune health. Previous studies examining the link between aspects of the social environment and biomarkers of immune health have relied heavily on one or two markers of inflammation, predominantly CRP and IL-6. By leveraging venous blood data from the HRS, we were able to examine indicators of cellular immunity and latent virus reactivation—a major innovation in studies of population immunosenescence. Second, many of the previous studies that have examined indicators of cellular immunity have been conducted in small, often clinical-based samples which limit generalizability of the findings. Using the HRS, data allowed us to examine these associations in a large US-based sample with broad generalizability to the US ageing population. Third, we were able to demonstrate empirically associations between indicators of the larger social environment that individuals occupy and subtle signatures of immune health. Such evidence continues to add to the growing body of work demonstrating how multiple levels of the social environment become embodied and produce the health outcome we see over the life course.

There are several limitations that should be considered when interpreting these results. While large and population-based, the HRS only captures those who survive to be enrolled in the HRS. It may be that those who have lived in the most disadvantaged environments and those who have the worst immune health did not survive to be enrolled in the HRS. However, we believe that such factors likely result in a bias toward the null since sicker individuals who may have also been more sensitive to the neighborhood environment through impacts on immune ageing, are more likely to die before entry into the HRS cohort. Further, those that participate in cohort studies are, in general, healthier than the average population and certainly represent those who are healthy enough to survive to be enrolled (63). This is also relevant when considering the associations among marginalized populations who, due to life course pressures of structural racism and oppression, may be systematically less likely to survive to be in the cohort study. With regards to how we measured race/ethnicity, we recognize that there is significant variation within race/ethnic categories as to how individuals may identify themselves. In addition, those captured in the “Other” race/ethnicity category likely represent a diverse set of experiences and though the small sample size precludes nuanced statistical investigation, we chose to represent this category in our analyzes. Given the complexity of capturing race/ethnicity status, appropriate caution should be used when interpreting findings with regards to race/ethnicity.

This study measured the neighborhood environment as a proxy for the social and economic conditions in which an individual of older age has likely lived for many years. But some individuals may have experienced moves due to changes in economic and social conditions which may be relevant to understand their immune health. Additionally, while our assessment of immune ageing is an innovation in the field, expanding beyond singular measures of inflammation, it captures only part of the complex immune dynamics at work. Our measures were also taken at a single point in time and do not capture the immune system as a whole, nor the ways in which it changes over time. These findings should be replicated in younger population cohorts and longitudinal studies, which may be less susceptible to selection effects found in older cohorts.

## Conclusion

The findings from this study provide initial evidence that large, nonspecific social exposures, such neighborhood socioeconomic conditions, may become embodied in cellular processes of immune ageing. This has implications for how we understand the drivers of immune ageing across the life course. Neighborhood environments, in contrast to other SDOH (i.e. education), are potentially modifiable exposures, and our results could be used to identify and address neighborhoods that may make residents particularly vulnerable to poor immune health. For example, federal policies, such as the Low Income Housing Tax Credit Program, that incentivize community investment and health-promoting affordable and subsidized housing may address long-standing trends in community disinvestment and associated health disparities (64–66). Such policies have already demonstrated improvements in social and physical housing and neighborhood conditions, as well as resident health; these health improvements also likely include immune health (67, 68). On a smaller scale, states and municipalities can prioritize placement of healthcare facilities and healthy food access points in neighborhoods that are particularly disadvantaged. While these interventions do not address the structural forces that have shaped where people can live and work, they may begin to address the inequity in how resources for health and wellbeing are spatially distributed. Our findings suggest that improving neighborhood conditions may have real implications for immune health, and importantly reduction of health inequities across the life course.

## Supplementary Material

pgae253_Supplementary_Data

## Data Availability

The Health and Retirement Study (HRS) data are available through a data use agreement with the HRS study. The National Neighborhood Data Archive (NaNDA) are publicly available and found at https://nanda.isr.umich.edu/ (https://doi.org/10.3886/ICPSR38528.v3).
