# Supplementary material for: The embodiment of the neighborhood socioeconomic environment in the architecture of the immune system
Source: PNAS Nexus. 2024 Jun 27;3(7):pgae253. doi: 10.1093/pnasnexus/pgae253 (PMC11244187; doi:10.1093/pnasnexus/pgae253)
Supplement: pgae253_Supplementary_Data [file pgae253_supplementary_data.docx]

**Figure S1.** Sample flow chart showing how the study sample was derived.

Total participants with complete and valid test results for the VBS:
**n = 9,933**

Total participants with valid measures for the CD8+: CD4+ Ratio
**n = 9,359**

Total participants with valid measures for the CD4+EMRA: Naïve Ratio
**n = 9,313**

Total participants with valid measures for the CD8+ EMRA: Naïve Ratio
**n = 9,314**

Total participants with valid measures for CMV
**n = 9,848**

Total participants with complete information on neighborhood, age, gender, race/ethnicity, income, and education
**n = 9,209**

Total participants with complete information on neighborhood, age, gender, race/ethnicity, income, and education
**n = 9,166**

Total participants with complete information on neighborhood, age, gender, race/ethnicity, income, and education
**n = 9,167**

Total participants with complete information on neighborhood, age, gender, race/ethnicity, income, and education
**n = 9,689**

Total participants with complete information on health behaviors and health status
**n = 9,114**

Total participants with complete information on health behaviors and health status
**n = 9,071**

Total participants with complete information on health behaviors and health status
**n = 9,072**

Total participants with complete information on health behaviors and health status
**n = 9,589**

Total participants with complete information CMV
**n = 9,072**

Total participants with complete information CMV
**n = 9,029**

Total participants with complete information CMV
**n = 9,030**

Final analytical sample
**n = 9,072**

Final analytical sample
**n = 9,029**

Final analytical sample
**n = 9,030**

Final analytical sample
**n = 9,589**

**Measure Details**

**Exposure: Neighborhood SES**

*Neighborhood disadvantage* is an analytically-derived index and is the mean of four variables from the American Community Survey ( 2012-2016 five year estimates): percent of the population with income below the federal poverty level, percent of households receiving public assistance income or food stamps, percent female-headed families, and percent of the population over the age of 16 that is unemployed.^30^ Mean scores range from 0-100. Neighborhoods that concentrate the socioeconomically disadvantaged tend to have fewer resources (e.g., healthy food stores, well-maintained parks, good schools, quality medical care) to promote good health^31,32^ and are often vulnerable to disinvestment and environmental hazards.^33^

*Neighborhood affluence* is distinct from the absence of disadvantage.^34^ It is the mean of three variables from the American Community Survey ( 2012-2016 five year estimates): percent of household with income greater than $75K per year, percent of the population over the age of 16 employed in professional or managerial occupations, and percent of the population with a Bachelor’s Degree or higher. Mean scores range from 0-100. Affluent neighborhoods are likely to attract a set of institutions (e.g., food stores, places to exercise, well-maintained buildings and parks) that foster a set of norms (e.g., an emphasis on exercise and healthy diets) conducive to good health.^35^ Distinct from simply being the converse of neighborhood disadvantage, neighborhood affluence is associated with higher levels of social control and leverage over local institutions that can foster social environments that facilitate health.^34^

**Other Neighborhood Covariates**

*Neighborhood Population Density* was also derived from the ACS (2012-2016 five-year estimates) and calculated as person per square miles in the census tract.

**Outcome: Biomarkers of Immune Function**

Immunophenotyping was performed on the entire VBS sample. Several recent studies describe in detailed the blood sample processing, laboratory assays, and the characterization of subtypes of T, B, monocytes, and natural killer (NK) cells.^17,18^ We focused on cell subtypes specific to CD4+ and CD8+ cells including: CD4+ (CD3+ CD19- CD8- CD4+), naïve CD4+ (CD45RA+ CD28- CD95-), terminally differentiated effector memory (EMRA) CD4+ (CD3+ CD19- CD8- CD4+ CD45RA+ CCR7- CD28-), CD8+ (CD3+ CD19- CD8+ CD4), Naïve CD8+ (CD3+ CD19- CD8+ CD4- CD45RA+ CCR7+ CD28+), EMRA CD8+ (CD3+ CD19- CD8+ CD4- CD45RA+ CCR7- CD28-). We then followed prior published approaches for creating measures of T cell immunosenescence in population-based research studies: ^24,36,37^

1. CD8+:CD4+
2. EMRA CD4+:Naïve CD4+
3. EMRA CD8+:Naïve CD8+.

For the primary analyses, we used a continuous percentage measure for each cell type. The percentages were defined in terms of the proportion of the parent cell population. For example, the percentage of naïve CD4+ T cells is defined as the proportion of naïve CD4+ T cells out of the total CD4+ cell parent population.

All ratio measures were standardized to facilitate interpretation of the coefficients and comparison across measures. For all three immune measures we constructed, higher values correspond to a more aged immune profile.

**Table S1.** Results of the regression analyses estimating the association between neighborhood disadvantage and the standardized CD8+:CD4+ ratio.

|  | Model1: Neighborhood Disadvantage + Age + Gender | | Model2: Neighborhood Disadvantage + Age + Gender + SES | | | Model3: Neighborhood Disadvantage + Age + Gender + SES + Race/Ethnicity | | Model4: Neighborhood Disadvantage + Age + Gender + SES + Race/Ethnicity + Health Behaviors and Chronic Conditions | | | Model5: Neighborhood Disadvantage + Age + Gender + SES + Race/Ethnicity + Health Behaviors and Chronic Conditions + Neighborhood Density | | | Model6: Neighborhood Disadvantage + Age + Gender + SES + Race/Ethnicity + Health Behaviors and Chronic Conditions + Neighborhood Density + CMV | |
| --- | --- | --- | --- | --- | --- | --- | --- | --- | --- | --- | --- | --- | --- | --- | --- |
|  | β | 95% CI | β | 95% CI | β | | 95% CI | | β | 95% CI | | β | 95% CI | β | 95% CI |
| **Neighborhood Characteristics** |  |  |  |  |  | |  | |  |  | |  |  |  |  |
| Disadvantage | -0.002 | [-.0071, .0035] | -0.003 | [-.0089, .0038] | -0.003 | | [-.0087, .0034] | | -0.004 | [-.0113, .0042] | | -0.004 | [-.0114, .0041] | -0.004 | [-.0128, .004] |
| Density |  |  |  |  |  | |  | |  |  | | 0 | [0, 0] | 0 | [0, 0] |
|  |  |  |  |  |  | |  | |  |  | |  |  |  |  |
| ***Demographics*** |  |  |  |  |  | |  | |  |  | |  |  |  |  |
| **Age** (years) | 0.0022 | [.0003, .0041] | 0.0019 | [.0004, .0034] | 0.0019 | | [.0005, .0034] | | 0.001 | [.0005, .0016] | | 0.001 | [.0005, .0016] | 0.0002 | [-.0008, .0013] |
| **Sex** (Female vs Male) | -0.059 | [-.1361, .0187] | -0.062 | [-.1441, .0208] | -0.062 | | [-.1441, .0208] | | -0.063 | [-.1477, .0212] | | -0.063 | [-.1478, .0211] | -0.074 | [-.1682, .0198] |
| **Race/ Ethnicity** |  |  |  |  |  | |  | |  |  | |  |  |  |  |
| Non-Hispanic Black |  |  |  |  | 0.004 | | [-.0096, .0176] | | -0.005 | [-.0328, .0234] | | -0.005 | [-.0335, .023] | -0.029 | [-.0769, .0197] |
| Hispanic |  |  |  |  | 0 | | [-.0178, .0178] | | 0.0039 | [-.0109, .0187] | | 0.0028 | [-.0121, .0176] | -0.026 | [-.0634, .0105] |
| Other Race/Ethnicity |  |  |  |  | 0.0047 | | [-.0295, .0388] | | 0.0057 | [-.0285, .0399] | | 0.0054 | [-.0289, .0396] | -0.021 | [-.077, .0341] |
| Non-Hispanic White (ref) |  |  |  |  |  | |  | |  |  | |  |  |  |  |
| **Education** |  |  |  |  |  | |  | |  |  | |  |  |  |  |
| Less than HS |  |  | 0.0227 | [-.0023, .0477] | 0.0226 | | [-.0067, .0519] | | 0.0218 | [-.0098, .0535] | | 0.022 | [-.0097, .0537] | 0.0093 | [-.0126, .0313] |
| HS Grad |  |  | 0.0399 | [-.0381, .118] | 0.0399 | | [-.0382, .1181] | | 0.0325 | [-.0331, .098] | | 0.0328 | [-.0328, .0984] | 0.0261 | [-.0335, .0857] |
| Some College |  |  | 0.0088 | [-.0079, .0255] | 0.0089 | | [-.0077, .0255] | | -0.002 | [-.0192, .0143] | | -0.002 | [-.0188, .0147] | -0.004 | [-.0213, .0133] |
| College Grad and Above (ref) |  |  |  |  |  | |  | |  |  | |  |  |  |  |
| **Household Income to Poverty Ratio** |  |  | -0.001 | [-.0027, .0006] | -0.001 | | [-.0028, .0007] | | -8E-04 | [-.0021, .0005] | | -8E-04 | [-.0021, .0005] | -8E-04 | [-.0022, .0005] |
|  |  |  |  |  |  | |  | |  |  | |  |  |  |  |
| ***Health Behaviors*** |  |  |  |  |  | |  | |  |  | |  |  |  |  |
| **Smoking Status** |  |  |  |  |  | |  | |  |  | |  |  |  |  |
| Current Smoker |  |  |  |  |  | |  | | -0.006 | [-.0274, .0148] | | -0.006 | [-.0275, .0147] | -0.014 | [-.0409, .0123] |
| Former Smoker |  |  |  |  |  | |  | | 0.0252 | [-.0256, .0761] | | 0.0252 | [-.0257, .076] | 0.0262 | [-.0253, .0778] |
| Never Smoker (ref) |  |  |  |  |  | |  | |  |  | |  |  |  |  |
| ***Health Status Indicators*** |  |  |  |  |  | |  | |  |  | |  |  |  |  |
| **Change in Self-Reported Health** |  |  |  |  |  | |  | | 0.0374 | [-.0419, .1166] | | 0.0374 | [-.0419, .1166] | 0.0384 | [-.0418, .1185] |
| **Self-Reported of a Change in Overall Health Status** |  |  |  |  |  | |  | |  |  | |  |  |  |  |
| Somewhat better |  |  |  |  |  | |  | | 0.1598 | [-.1402, .4597] | | 0.1598 | [-.1402, .4597] | 0.1567 | [-.1409, .4542] |
| Somewhat worse |  |  |  |  |  | |  | | -0.044 | [-.1372, .0495] | | -0.044 | [-.1373, .0495] | -0.044 | [-.1382, .0494] |
| Same (ref) |  |  |  |  |  | |  | |  |  | |  |  |  |  |
| **Chronic Conditions Index** |  |  |  |  |  | |  | | 0.0348 | [-.0287, .0984] | | 0.0348 | [-.0287, .0984] | 0.0348 | [-.029, .0986] |
| **Change in Functional Limitations** |  |  |  |  |  | |  | | -0.004 | [-.0135, .0048] | | -0.004 | [-.0136, .0047] | -0.004 | [-.013, .0047] |
|  |  |  |  |  |  | |  | |  |  | |  |  |  |  |
| **CMV** |  |  |  |  |  | |  | |  |  | |  |  |  |  |
| CMV IgG Continuous Antibodies U/mL of blood |  |  |  |  |  | |  | |  |  | |  |  | 0.0126 | [.0013, .0239] |
| **n = 9,072** |  |  |  |  |  | |  | |  |  | |  |  |  |  |

**Table S2.** Results of the regression analyses estimating the association between neighborhood affluence and the standardized CD8+:CD4+ ratio.

|  | Model1: Neighborhood Affluence + Age + Gender | | Model2: Neighborhood Affluence + Age + Gender + SES | | Model3: Neighborhood Affluence + Age + Gender + SES + Race/Ethnicity | | Model4: Neighborhood Affluence + Age + Gender + SES + Race/Ethnicity + Health Behaviors and Chronic Conditions | | Model5: Neighborhood Affluence + Age + Gender + SES + Race/Ethnicity + Health Behaviors and Chronic Conditions + Neighborhood Density | | Model6: Neighborhood Affluence + Age + Gender + SES + Race/Ethnicity + Health Behaviors and Chronic Conditions + Neighborhood Density + CMV | |
| --- | --- | --- | --- | --- | --- | --- | --- | --- | --- | --- | --- | --- |
|  | β | 95% CI | β | 95% CI | β | 95% CI | β | 95% CI | β | 95% CI | β | 95% CI |
| **Neighborhood Characteristics** |  |  |  |  |  |  |  |  |  |  |  |  |
| Affluence | 0.0006 | [-.001, .0021] | 0.0011 | [-.0013, .0036] | 0.0011 | [-.0012, .0034] | 0.0015 | [-.0015, .0044] | 0.0015 | [-.0015, .0045] | 0.0018 | [-.0015, .0051] |
| Density |  |  |  |  |  |  |  |  | 0 | [0, 0] | 0 | [0, 0] |
|  |  |  |  |  |  |  |  |  |  |  |  |  |
| ***Demographics*** |  |  |  |  |  |  |  |  |  |  |  |  |
| **Age** (years) | 0.0023 | [.0001, .0044] | 0.002 | [.0003, .0037] | 0.002 | [.0004, .0035] | 0.0011 | [.0006, .0016] | 0.0011 | [.0006, .0016] | 0.0003 | [-.0007, .0012] |
| **Sex** (Female vs Male) | -0.059 | [-.1371, .0193] | -0.062 | [-.1466, .0217] | -0.062 | [-.1465, .0217] | -0.064 | [-.151, .0223] | -0.064 | [-.1508, .0222] | -0.076 | [-.172, .0209] |
| **Race/ Ethnicity** |  |  |  |  |  |  |  |  |  |  |  |  |
| Non-Hispanic Black |  |  |  |  | -0.004 | [-.0359, .0277] | -0.016 | [-.0701, .0373] | -0.016 | [-.066, .0349] | -0.041 | [-.1152, .0324] |
| Hispanic |  |  |  |  | -0.004 | [-.0307, .023] | -0.001 | [-.0257, .0229] | -1E-04 | [-.0201, .0198] | -0.03 | [-.0759, .015] |
| Other Race/Ethnicity |  |  |  |  | 0.0016 | [-.0389, .0422] | 0.0015 | [-.0404, .0433] | 0.0018 | [-.0388, .0424] | -0.026 | [-.0904, .0379] |
| Non-Hispanic White (ref) |  |  |  |  |  |  |  |  |  |  |  |  |
| **Education** |  |  |  |  |  |  |  |  |  |  |  |  |
| Less than HS |  |  | 0.0287 | [-.0043, .0617] | 0.0301 | [-.0121, .0723] | 0.0311 | [-.0162, .0784] | 0.0312 | [-.0165, .079] | 0.0206 | [-.0179, .0592] |
| HS Grad |  |  | 0.0469 | [-.0445, .1383] | 0.047 | [-.0449, .1389] | 0.0416 | [-.0412, .1244] | 0.0414 | [-.0406, .1235] | 0.0366 | [-.0411, .1143] |
| Some College |  |  | 0.0149 | [-.0123, .042] | 0.0149 | [-.0118, .0415] | 0.0054 | [-.0111, .0219] | 0.0051 | [-.011, .0213] | 0.0048 | [-.0106, .0201] |
| College Grad and Above (ref) |  |  |  |  |  |  |  |  |  |  |  |  |
| **Household Income to Poverty Ratio** |  |  | -0.001 | [-.0032, .0007] | -0.001 | [-.0033, .0008] | -0.001 | [-.0028, .0007] | -0.001 | [-.0028, .0007] | -0.001 | [-.0029, .0007] |
|  |  |  |  |  |  |  |  |  |  |  |  |  |
| ***Health Behaviors*** |  |  |  |  |  |  |  |  |  |  |  |  |
| **Smoking Status** |  |  |  |  |  |  |  |  |  |  |  |  |
| Current Smoker |  |  |  |  |  |  | -0.006 | [-.0259, .0146] | -0.006 | [-.0255, .0144] | -0.013 | [-.0387, .012] |
| Former Smoker |  |  |  |  |  |  | 0.0251 | [-.0255, .0758] | 0.0252 | [-.0257, .076] | 0.0262 | [-.0253, .0778] |
| Never Smoker (ref) |  |  |  |  |  |  |  |  |  |  |  |  |
| ***Health Status Indicators*** |  |  |  |  |  |  |  |  |  |  |  |  |
| **Change in Self-Reported Health** |  |  |  |  |  |  | 0.0373 | [-.0418, .1164] | 0.0373 | [-.0418, .1164] | 0.0383 | [-.0417, .1183] |
| **Self-Reported of a Change in Overall Health Status** |  |  |  |  |  |  |  |  |  |  |  |  |
| Somewhat better |  |  |  |  |  |  | 0.1597 | [-.14, .4594] | 0.1597 | [-.14, .4594] | 0.1565 | [-.1407, .4538] |
| Somewhat worse |  |  |  |  |  |  | -0.044 | [-.1387, .0501] | -0.044 | [-.1381, .0498] | -0.045 | [-.1392, .0498] |
| Same (ref) |  |  |  |  |  |  |  |  |  |  |  |  |
| **Chronic Conditions Index** |  |  |  |  |  |  | 0.0353 | [-.029, .0995] | 0.0353 | [-.0291, .0996] | 0.0354 | [-.0293, .1001] |
| **Change in Functional Limitations** |  |  |  |  |  |  | -0.004 | [-.0132, .0046] | -0.004 | [-.0131, .0046] | -0.004 | [-.0124, .0045] |
|  |  |  |  |  |  |  |  |  |  |  |  |  |
| **CMV** |  |  |  |  |  |  |  |  |  |  |  |  |
| CMV IgG Continuous Antibodies U/mL of blood |  |  |  |  |  |  |  |  |  |  | 0.0128 | [.0012, .0245] |
| **n = 9,072** |  |  |  |  |  |  |  |  |  |  |  |  |

**Table S3.** Results of the regression analyses estimating the association between neighborhood disadvantage and the standardized CD4+ EMRA: Naïve ratio.

|  | Model1: Neighborhood Disadvantage + Age + Gender | | Model2: Neighborhood Disadvantage + Age + Gender + SES | | Model3: Neighborhood Disadvantage + Age + Gender + SES + Race/Ethnicity | | Model4: Neighborhood Disadvantage + Age + Gender + SES + Race/Ethnicity + Health Behaviors and Chronic Conditions | | Model5: Neighborhood Disadvantage + Age + Gender + SES + Race/Ethnicity + Health Behaviors and Chronic Conditions + Neighborhood Density | | Model6: Neighborhood Disadvantage + Age + Gender + SES + Race/Ethnicity + Health Behaviors and Chronic Conditions + Neighborhood Density + CMV | |
| --- | --- | --- | --- | --- | --- | --- | --- | --- | --- | --- | --- | --- |
|  | β | 95% CI | β | 95% CI | β | 95% CI | β | 95% CI | β | 95% CI | β | 95% CI |
| **Neighborhood Characteristics** |  |  |  |  |  |  |  |  |  |  |  |  |
| Disadvantage | 0.0053 | [.0003, .0104] | 0.0046 | [.0001, .009] | 0.0033 | [-.0012, .0078] | 0.0033 | [-.0016, .0081] | 0.0032 | [-.0019, .0084] | 0.0026 | [-.0028, .0079] |
| Density |  |  |  |  |  |  |  |  | 0 | [0, 0] | 0 | [0, 0] |
|  |  |  |  |  |  |  |  |  |  |  |  |  |
| ***Demographics*** |  |  |  |  |  |  |  |  |  |  |  |  |
| **Age** (years) | 0.0045 | [.0015, .0075] | 0.0044 | [.0012, .0076] | 0.0046 | [.0013, .0078] | 0.0047 | [.0014, .008] | 0.0047 | [.0014, .008] | 0.004 | [.0008, .0072] |
| **Sex** (Female vs Male) | -0.023 | [-.0606, .0154] | -0.025 | [-.0618, .0121] | -0.025 | [-.0614, .0121] | -0.027 | [-.0631, .0089] | -0.027 | [-.063, .0087] | -0.037 | [-.0724, -.0022] |
| **Race/ Ethnicity** |  |  |  |  |  |  |  |  |  |  |  |  |
| Non-Hispanic Black |  |  |  |  | 0.0458 | [.0031, .0885] | 0.0462 | [.0031, .0893] | 0.0457 | [.005, .0863] | 0.0245 | [-.0158, .0649] |
| Hispanic |  |  |  |  | 0.0505 | [-.0192, .1201] | 0.0507 | [-.018, .1193] | 0.0495 | [-.025, .124] | 0.0227 | [-.0559, .1013] |
| Other Race/Ethnicity |  |  |  |  | -0.022 | [-.0393, -.005] | -0.023 | [-.0401, -.0057] | -0.023 | [-.0407, -.0057] | -0.048 | [-.0702, -.0252] |
| Non-Hispanic White (ref) |  |  |  |  |  |  |  |  |  |  |  |  |
| **Education** |  |  |  |  |  |  |  |  |  |  |  |  |
| Less than HS |  |  | 0.0563 | [-.0003, .1128] | 0.0413 | [-.0058, .0884] | 0.0411 | [-.0089, .0912] | 0.0413 | [-.0086, .0911] | 0.0301 | [-.0216, .0818] |
| HS Grad |  |  | 0.0063 | [-.0236, .0362] | 0.0045 | [-.0257, .0348] | 0.0058 | [-.0268, .0383] | 0.0061 | [-.0269, .0391] | -6E-04 | [-.0336, .0325] |
| Some College |  |  | 0.0822 | [-.0702, .2346] | 0.0815 | [-.0709, .2338] | 0.0805 | [-.0747, .2358] | 0.0809 | [-.0755, .2373] | 0.0789 | [-.0771, .2348] |
| College Grad and Above (ref) |  |  |  |  |  |  |  |  |  |  |  |  |
| **Household Income to Poverty Ratio** |  |  | -1E-04 | [-.0011, .001] | 0.0001 | [-.001, .0011] | 0.0001 | [-.0009, .001] | 0.0001 | [-.0009, .001] | 0 | [-.0009, .001] |
|  |  |  |  |  |  |  |  |  |  |  |  |  |
| ***Health Behaviors*** |  |  |  |  |  |  |  |  |  |  |  |  |
| **Smoking Status** |  |  |  |  |  |  |  |  |  |  |  |  |
| Current Smoker |  |  |  |  |  |  | -0.006 | [-.0378, .0261] | -0.006 | [-.038, .0262] | -0.014 | [-.0471, .0197] |
| Former Smoker |  |  |  |  |  |  | -0.01 | [-.0538, .0336] | -0.01 | [-.0541, .0337] | -0.009 | [-.0532, .0345] |
| Never Smoker (ref) |  |  |  |  |  |  |  |  |  |  |  |  |
| ***Health Status Indicators*** |  |  |  |  |  |  |  |  |  |  |  |  |
| **Change in Self-Reported Health** |  |  |  |  |  |  | -0.01 | [-.0213, .001] | -0.01 | [-.0213, .001] | -0.009 | [-.0202, .0019] |
| **Self-Reported of a Change in Overall Health Status** |  |  |  |  |  |  |  |  |  |  |  |  |
| Somewhat better |  |  |  |  |  |  | 0.0121 | [-.0195, .0437] | 0.0121 | [-.0195, .0437] | 0.0083 | [-.0238, .0404] |
| Somewhat worse |  |  |  |  |  |  | 0.0164 | [-.0431, .0758] | 0.0162 | [-.0431, .0756] | 0.0157 | [-.0438, .0752] |
| Same (ref) |  |  |  |  |  |  |  |  |  |  |  |  |
| **Chronic Conditions Index** |  |  |  |  |  |  | -0.001 | [-.0154, .0125] | -0.001 | [-.0153, .0124] | -0.002 | [-.0155, .0124] |
| **Change in Functional Limitations** |  |  |  |  |  |  | -7E-04 | [-.0213, .02] | -7E-04 | [-.0216, .0201] | -5E-04 | [-.0215, .0204] |
|  |  |  |  |  |  |  |  |  |  |  |  |  |
| **CMV** |  |  |  |  |  |  |  |  |  |  |  |  |
| CMV IgG Continuous Antibodies U/mL of blood |  |  |  |  |  |  |  |  |  |  | 0.0115 | [.0075, .0155] |
| **n = 9,029** |  |  |  |  |  |  |  |  |  |  |  |  |

**Table S4.** Results of the regression analyses estimating the association between neighborhood affluence and the standardized CD4+ EMRA: Naïve ratio.

|  | Model1: Neighborhood Affluence + Age + Gender | | Model2: Neighborhood Affluence + Age + Gender + SES | | Model3: Neighborhood Affluence + Age + Gender + SES + Race/Ethnicity | | Model4: Neighborhood Affluence + Age + Gender + SES + Race/Ethnicity + Health Behaviors and Chronic Conditions | | Model5: Neighborhood Affluence + Age + Gender + SES + Race/Ethnicity + Health Behaviors and Chronic Conditions + Neighborhood Density | | Model6: Neighborhood Affluence + Age + Gender + SES + Race/Ethnicity + Health Behaviors and Chronic Conditions + Neighborhood Density + CMV | |
| --- | --- | --- | --- | --- | --- | --- | --- | --- | --- | --- | --- | --- |
|  | β | 95% CI | β | 95% CI | β | 95% CI | β | 95% CI | β | 95% CI | β | 95% CI |
| **Neighborhood Characteristics** |  |  |  |  |  |  |  |  |  |  |  |  |
| Affluence | -0.001 | [-.0023, .0004] | -0.0005 | [-.0018, .0007] | -0.0002 | [-.0014, .001] | -0.0002 | [-.0015, .0011] | -0.0002 | [-.0015, .001] | 0.0001 | [-.0013, .0014] |
| Density |  |  |  |  |  |  |  |  | 0 | [0, 0] | 0 | [0, 0] |
|  |  |  |  |  |  |  |  |  |  |  |  |  |
| ***Demographics*** |  |  |  |  |  |  |  |  |  |  |  |  |
| **Age** (years) | 0.0043 | [.0013, .0073] | 0.0042 | [.0011, .0073] | 0.0045 | [.0013, .0077] | 0.0046 | [.0013, .0078] | 0.0046 | [.0013, .0078] | 0.0039 | [.0007, .007] |
| **Sex** (Female vs Male) | -0.021 | [-.0588, .0159] | -0.024 | [-.0604, .0123] | -0.0242 | [-.0604, .012] | -0.0266 | [-.062, .0088] | -0.0267 | [-.062, .0086] | -0.0373 | [-.072, -.0026] |
| **Race/ Ethnicity** |  |  |  |  |  |  |  |  |  |  |  |  |
| Non-Hispanic Black |  |  |  |  | 0.0649 | [.0332, .0967] | 0.0652 | [.0333, .0971] | 0.0632 | [.0323, .0941] | 0.0397 | [.0067, .0728] |
| Hispanic |  |  |  |  | 0.0621 | [-.0197, .1438] | 0.0627 | [-.0189, .1443] | 0.0596 | [-.0275, .1467] | 0.0318 | [-.0588, .1224] |
| Other Race/Ethnicity |  |  |  |  | -0.0174 | [-.0342, -.0006] | -0.018 | [-.0354, -.0007] | -0.0189 | [-.0373, -.0005] | -0.0446 | [-.0691, -.02] |
| Non-Hispanic White (ref) |  |  |  |  |  |  |  |  |  |  |  |  |
| **Education** |  |  |  |  |  |  |  |  |  |  |  |  |
| Less than HS |  |  | 0.0694 | [.0072, .1316] | 0.0473 | [.0002, .0945] | 0.0469 | [-.0029, .0967] | 0.0466 | [-.0033, .0966] | 0.0371 | [-.0141, .0883] |
| HS Grad |  |  | 0.0082 | [-.021, .0375] | 0.0062 | [-.0233, .0358] | 0.0073 | [-.0246, .0393] | 0.0078 | [-.0244, .0399] | 0.0027 | [-.0293, .0347] |
| Some College |  |  | 0.0813 | [-.0764, .239] | 0.0815 | [-.0761, .2391] | 0.0804 | [-.0804, .2412] | 0.0811 | [-.0803, .2425] | 0.0804 | [-.0809, .2417] |
| College Grad and Above (ref) |  |  |  |  |  |  |  |  |  |  |  |  |
| **Household Income to Poverty Ratio** |  |  | -0.0002 | [-.0014, .001] | 0 | [-.0012, .0011] | 0 | [-.0011, .0011] | 0 | [-.0011, .0011] | -0.0001 | [-.0012, .001] |
|  |  |  |  |  |  |  |  |  |  |  |  |  |
| ***Health Behaviors*** |  |  |  |  |  |  |  |  |  |  |  |  |
| **Smoking Status** |  |  |  |  |  |  |  |  |  |  |  |  |
| Current Smoker |  |  |  |  |  |  | -0.0042 | [-.036, .0276] | -0.0044 | [-.0365, .0277] | -0.0122 | [-.0455, .0212] |
| Former Smoker |  |  |  |  |  |  | -0.01 | [-.0538, .0337] | -0.0102 | [-.0541, .0337] | -0.0094 | [-.0532, .0345] |
| Never Smoker (ref) |  |  |  |  |  |  |  |  |  |  |  |  |
| ***Health Status Indicators*** |  |  |  |  |  |  |  |  |  |  |  |  |
| **Change in Self-Reported Health** |  |  |  |  |  |  | -0.01 | [-.0212, .0012] | -0.01 | [-.0212, .0012] | -0.009 | [-.0201, .002] |
| **Self-Reported of a Change in Overall Health Status** |  |  |  |  |  |  |  |  |  |  |  |  |
| Somewhat better |  |  |  |  |  |  | 0.0127 | [-.0188, .0442] | 0.0127 | [-.0188, .0442] | 0.0088 | [-.0232, .0408] |
| Somewhat worse |  |  |  |  |  |  | 0.0171 | [-.0422, .0764] | 0.0168 | [-.0424, .076] | 0.0162 | [-.0431, .0756] |
| Same (ref) |  |  |  |  |  |  |  |  |  |  |  |  |
| **Chronic Conditions Index** |  |  |  |  |  |  | -0.0008 | [-.0147, .0132] | -0.0009 | [-.0148, .013] | -0.0009 | [-.0149, .0131] |
| **Change in Functional Limitations** |  |  |  |  |  |  | -0.0011 | [-.0214, .0193] | -0.0011 | [-.0215, .0193] | -0.0009 | [-.0214, .0196] |
|  |  |  |  |  |  |  |  |  |  |  |  |  |
| **CMV** |  |  |  |  |  |  |  |  |  |  |  |  |
| CMV IgG Continuous Antibodies U/mL of blood |  |  |  |  |  |  |  |  |  |  | 0.0117 | [.0077, .0158] |
| **n = 9,029** |  |  |  |  |  |  |  |  |  |  |  |  |

**Table S5.** Results of the regression analyses estimating the association between neighborhood disadvantage and the standardized CD8+ EMRA: Naïve ratio.

|  | Model1: Neighborhood Disadvantage + Age + Gender | | Model2: Neighborhood Disadvantage + Age + Gender + SES | | Model3: Neighborhood Disadvantage + Age + Gender + SES + Race/Ethnicity | | Model4: Neighborhood Disadvantage + Age + Gender + SES + Race/Ethnicity + Health Behaviors and Chronic Conditions | | Model5: Neighborhood Disadvantage + Age + Gender + SES + Race/Ethnicity + Health Behaviors and Chronic Conditions + Neighborhood Density | | Model6: Neighborhood Disadvantage + Age + Gender + SES + Race/Ethnicity + Health Behaviors and Chronic Conditions + Neighborhood Density + CMV | |
| --- | --- | --- | --- | --- | --- | --- | --- | --- | --- | --- | --- | --- |
|  | β | 95% CI | β | 95% CI | β | 95% CI | β | 95% CI | β | 95% CI | β | 95% CI |
| **Neighborhood Characteristics** |  |  |  |  |  |  |  |  |  |  |  |  |
| Disadvantage | 0.003 | [-.0018, .0068] | 0.002 | [-.0028, .0057] | 0.0029 | [-.0016, .0074] | 0.0027 | [-.002, .0074] | 0.0029 | [-.002, .0077] | 0.0021 | [-.0027, .0069] |
| Density |  |  |  |  |  |  |  |  | 0 | [0, 0] | 0 | [0, 0] |
|  |  |  |  |  |  |  |  |  |  |  |  |  |
| ***Demographics*** |  |  |  |  |  |  |  |  |  |  |  |  |
| **Age** (years) | 0.012 | [.0086, .0155] | 0.012 | [.0083, .0153] | 0.0118 | [.0083, .0153] | 0.0111 | [.0078, .0144] | 0.0111 | [.0078, .0144] | 0.0103 | [.0071, .0136] |
| **Sex** (Female vs Male) | -0.074 | [-.1214, -.0266] | -0.076 | [-.122, -.0301] | -0.076 | [-.122, -.03] | -0.081 | [-.1298, -.0315] | -0.081 | [-.1297, -.0314] | -0.092 | [-.142, -.0413] |
| **Race/ Ethnicity** |  |  |  |  |  |  |  |  |  |  |  |  |
| Non-Hispanic Black |  |  |  |  | -0.07 | [-.1246, -.0157] | -0.074 | [-.1306, -.0178] | -0.073 | [-.1296, -.0161] | -0.097 | [-.1546, -.0386] |
| Hispanic |  |  |  |  | -0.009 | [-.0691, .0519] | -0.01 | [-.0728, .052] | -0.007 | [-.0722, .0573] | -0.037 | [-.1039, .0297] |
| Other Race/Ethnicity |  |  |  |  | 0.0345 | [-.125, .1939] | 0.0334 | [-.1298, .1966] | 0.0342 | [-.1294, .1978] | 0.007 | [-.1584, .1724] |
| Non-Hispanic White (ref) |  |  |  |  |  |  |  |  |  |  |  |  |
| **Education** |  |  |  |  |  |  |  |  |  |  |  |  |
| Less than HS |  |  | 0.072 | [-.0189, .1633] | 0.0761 | [-.0182, .1703] | 0.0777 | [-.0215, .1769] | 0.0774 | [-.022, .1767] | 0.065 | [-.0371, .1671] |
| HS Grad |  |  | 0.019 | [-.0388, .0768] | 0.0202 | [-.038, .0784] | 0.0197 | [-.0388, .0782] | 0.0188 | [-.04, .0777] | 0.0114 | [-.049, .0718] |
| Some College |  |  | 0.056 | [-.0305, .1426] | 0.0559 | [-.0306, .1425] | 0.0544 | [-.0332, .1419] | 0.0534 | [-.0343, .1411] | 0.0509 | [-.0369, .1388] |
| College Grad and Above (ref) |  |  |  |  |  |  |  |  |  |  |  |  |
| **Household Income to Poverty Ratio** |  |  | 3E-04 | [-.0019, .0026] | 0.0002 | [-.002, .0025] | 0.0003 | [-.0018, .0025] | 0.0003 | [-.0018, .0025] | 0.0003 | [-.0019, .0025] |
|  |  |  |  |  |  |  |  |  |  |  |  |  |
| ***Health Behaviors*** |  |  |  |  |  |  |  |  |  |  |  |  |
| **Smoking Status** |  |  |  |  |  |  |  |  |  |  |  |  |
| Current Smoker |  |  |  |  |  |  | -0.045 | [-.1079, .0178] | -0.045 | [-.1079, .018] | -0.054 | [-.1178, .0104] |
| Former Smoker |  |  |  |  |  |  | -0.012 | [-.0627, .0396] | -0.011 | [-.0626, .0398] | -0.011 | [-.0617, .0408] |
| Never Smoker (ref) |  |  |  |  |  |  |  |  |  |  |  |  |
| ***Health Status Indicators*** |  |  |  |  |  |  |  |  |  |  |  |  |
| **Change in Self-Reported Health** |  |  |  |  |  |  | -0.02 | [-.051, .012] | -0.02 | [-.0511, .012] | -0.019 | [-.05, .0131] |
| **Self-Reported of a Change in Overall Health Status** |  |  |  |  |  |  |  |  |  |  |  |  |
| Somewhat better |  |  |  |  |  |  | 0.0218 | [-.0494, .093] | 0.0218 | [-.0494, .093] | 0.0174 | [-.0539, .0887] |
| Somewhat worse |  |  |  |  |  |  | -0.004 | [-.0639, .0551] | -0.004 | [-.0635, .0552] | -0.005 | [-.0644, .0546] |
| Same (ref) |  |  |  |  |  |  |  |  |  |  |  |  |
| **Chronic Conditions Index** |  |  |  |  |  |  | 0.0152 | [-.0025, .033] | 0.0153 | [-.0025, .0331] | 0.0153 | [-.0025, .0332] |
| **Change in Functional Limitations** |  |  |  |  |  |  | 0.0028 | [-.0302, .0358] | 0.0029 | [-.0302, .036] | 0.0031 | [-.0301, .0363] |
|  |  |  |  |  |  |  |  |  |  |  |  |  |
| **CMV** |  |  |  |  |  |  |  |  |  |  |  |  |
| CMV IgG Continuous Antibodies U/mL of blood |  |  |  |  |  |  |  |  |  |  | 0.0127 | [.0068, .0186] |
| **n = 9,030** |  |  |  |  |  |  |  |  |  |  |  |  |

**Table S6.** Results of the regression analyses estimating the association between neighborhood affluence and the standardized CD8+ EMRA: Naïve ratio.

|  | Model1: Neighborhood Affluence + Age + Gender | | Model2: Neighborhood Affluence + Age + Gender + SES | | Model3: Neighborhood Affluence + Age + Gender + SES + Race/Ethnicity | | Model4: Neighborhood Affluence + Age + Gender + SES + Race/Ethnicity + Health Behaviors and Chronic Conditions | | Model5: Neighborhood Affluence + Age + Gender + SES + Race/Ethnicity + Health Behaviors and Chronic Conditions + Neighborhood Density | | Model6: Neighborhood Affluence + Age + Gender + SES + Race/Ethnicity + Health Behaviors and Chronic Conditions + Neighborhood Density + CMV | | |
| --- | --- | --- | --- | --- | --- | --- | --- | --- | --- | --- | --- | --- | --- |
|  | β | 95% CI | β | 95% CI | β | 95% CI | β | 95% CI | β | 95% CI | β | 95% CI |  |
| **Neighborhood Characteristics** |  |  |  |  |  |  |  |  |  |  |  |  |  |
| Affluence | -6E-04 | [-.0021, .0009] | -1E-04 | [-.0019, .0017] | -3E-04 | [-.0021, .0015] | -2E-04 | [-.0019, .0016] | -2E-04 | [-.0019, .0016] | 0.0002 | [-.0016, .0019] |  |
| Density |  |  |  |  |  |  |  |  | 0 | [0, 0] | 0 | [0, 0] |  |
|  |  |  |  |  |  |  |  |  |  |  |  |  |  |
| ***Demographics*** |  |  |  |  |  |  |  |  |  |  |  |  |  |
| **Age** (years) | 0.012 | [.0085, .0154] | 0.0118 | [.0083, .0152] | 0.0117 | [.0082, .0152] | 0.011 | [.0077, .0143] | 0.011 | [.0077, .0143] | 0.0102 | [.007, .0135] |  |
| **Sex** (Female vs Male) | -0.074 | [-.1205, -.0265] | -0.076 | [-.1216, -.03] | -0.076 | [-.1213, -.0297] | -0.08 | [-.1292, -.0313] | -0.08 | [-.1292, -.0312] | -0.092 | [-.142, -.0414] |  |
| **Race/ Ethnicity** |  |  |  |  |  |  |  |  |  |  |  |  |  |
| Non-Hispanic Black |  |  |  |  | -0.054 | [-.1046, -.0031] | -0.058 | [-.1111, -.0054] | -0.057 | [-.1111, -.0026] | -0.083 | [-.1385, -.0278] |  |
| Hispanic |  |  |  |  | 0.0012 | [-.06, .0624] | -3E-04 | [-.063, .0624] | 0.002 | [-.0634, .0673] | -0.029 | [-.0964, .0387] |  |
| Other Race/Ethnicity |  |  |  |  | 0.0386 | [-.1194, .1967] | 0.0375 | [-.1241, .1991] | 0.0381 | [-.1241, .2003] | 0.0097 | [-.1546, .1739] |  |
| Non-Hispanic White (ref) |  |  |  |  |  |  |  |  |  |  |  |  |  |
| **Education** |  |  |  |  |  |  |  |  |  |  |  |  |  |
| Less than HS |  |  | 0.0772 | [-.0232, .1777] | 0.0803 | [-.0225, .1832] | 0.0826 | [-.0245, .1898] | 0.0828 | [-.0243, .1899] | 0.0723 | [-.0371, .1818] |  |
| HS Grad |  |  | 0.0201 | [-.0422, .0824] | 0.021 | [-.0416, .0836] | 0.0211 | [-.0417, .0838] | 0.0207 | [-.0421, .0836] | 0.0151 | [-.049, .0791] |  |
| Some College |  |  | 0.0561 | [-.0336, .1458] | 0.0555 | [-.0342, .1452] | 0.0543 | [-.036, .1447] | 0.0538 | [-.0365, .1442] | 0.0529 | [-.0375, .1433] |  |
| College Grad and Above (ref) |  |  |  |  |  |  |  |  |  |  |  |  |  |
| **Household Income to Poverty Ratio** |  |  | 0.0002 | [-.002, .0025] | 0.0002 | [-.002, .0024] | 0.0003 | [-.0019, .0024] | 0.0003 | [-.0019, .0024] | 0.0002 | [-.002, .0024] |  |
|  |  |  |  |  |  |  |  |  |  |  |  |  |  |
| ***Health Behaviors*** |  |  |  |  |  |  |  |  |  |  |  |  |  |
| **Smoking Status** |  |  |  |  |  |  |  |  |  |  |  |  |  |
| Current Smoker |  |  |  |  |  |  | -0.044 | [-.1057, .0184] | -0.044 | [-.1058, .0188] | -0.052 | [-.1158, .0113] |  |
| Former Smoker |  |  |  |  |  |  | -0.012 | [-.0627, .0397] | -0.011 | [-.0627, .0398] | -0.011 | [-.0618, .0408] |  |
| Never Smoker (ref) |  |  |  |  |  |  |  |  |  |  |  |  |  |
| ***Health Status Indicators*** |  |  |  |  |  |  |  |  |  |  |  |  |  |
| **Change in Self-Reported Health** |  |  |  |  |  |  | -0.019 | [-.0509, .0121] | -0.019 | [-.0509, .0121] | -0.018 | [-.0499, .0132] |  |
| **Self-Reported of a Change in Overall Health Status** |  |  |  |  |  |  |  |  |  |  |  |  |  |
| Somewhat better |  |  |  |  |  |  | 0.0223 | [-.0489, .0935] | 0.0223 | [-.0489, .0935] | 0.0179 | [-.0534, .0892] |  |
| Somewhat worse |  |  |  |  |  |  | -0.004 | [-.063, .0554] | -0.004 | [-.0627, .0556] | -0.004 | [-.0637, .0549] |  |
| Same (ref) |  |  |  |  |  |  |  |  |  |  |  |  |  |
| **Chronic Conditions Index** |  |  |  |  |  |  | 0.0158 | [-.0019, .0334] | 0.0159 | [-.0018, .0335] | 0.016 | [-.0018, .0338] |  |
| **Change in Functional Limitations** |  |  |  |  |  |  | 0.0025 | [-.0305, .0354] | 0.0025 | [-.0305, .0355] | 0.0028 | [-.0303, .0358] |  |
|  |  |  |  |  |  |  |  |  |  |  |  |  |  |
| **CMV** |  |  |  |  |  |  |  |  |  |  |  |  |  |
| CMV IgG Continuous Antibodies U/mL of blood |  |  |  |  |  |  |  |  |  |  | 0.013 | [.0071, .0189] |  |
| **n = 9,030** |  |  |  |  |  |  |  |  |  |  |  |  |  |

**Table S7.** Results of the regression analyses estimating the association between neighborhood disadvantage and the standardized CMV IgG.

|  | Model1: Neighborhood Disadvantage + Age + Gender | | Model2: Neighborhood Disadvantage + Age + Gender + SES | | Model3: Neighborhood Disadvantage + Age + Gender + SES + Race/Ethnicity | | Model4: Neighborhood Disadvantage + Age + Gender + SES + Race/Ethnicity + Health Behaviors and Chronic Conditions | | Model5: Neighborhood Disadvantage + Age + Gender + SES + Race/Ethnicity + Health Behaviors and Chronic Conditions + Neighborhood Density | |
| --- | --- | --- | --- | --- | --- | --- | --- | --- | --- | --- |
|  | β | 95% CI | β | 95% CI | β | 95% CI | β | 95% CI | β | 95% CI |
| **Neighborhood Characteristics** |  |  |  |  |  |  |  |  |  |  |
| Disadvantage | 0.029 | [.0242, .0334] | 0.0217 | [.0169, .0266] | 0.012 | [.0066, .0175] | 0.011 | [.0056, .0165] | 0.0107 | [.0052, .0163] |
| Density |  |  |  |  |  |  |  |  | 0 | [0, 0] |
|  |  |  |  |  |  |  |  |  |  |  |
| ***Demographics*** |  |  |  |  |  |  |  |  |  |  |
| **Age** (years) | 0.007 | [.0045, .01] | 0.005 | [.0023, .0077] | 0.0065 | [.0037, .0092] | 0.0077 | [.0047, .0106] | 0.0076 | [.0047, .0106] |
| **Sex** (Female vs Male) | 0.275 | [.225, .3247] | 0.263 | [.2135, .3126] | 0.2641 | [.2151, .3132] | 0.2694 | [.219, .3199] | 0.2692 | [.2188, .3197] |
| **Race/ Ethnicity** |  |  |  |  |  |  |  |  |  |  |
| Non-Hispanic Black |  |  |  |  | 0.3603 | [.2609, .4597] | 0.3396 | [.2391, .4401] | 0.3368 | [.2369, .4368] |
| Hispanic |  |  |  |  | 0.3029 | [.2117, .3941] | 0.3273 | [.2356, .4191] | 0.3212 | [.2289, .4135] |
| Other Race/Ethnicity |  |  |  |  | 0.2251 | [.0991, .3512] | 0.2374 | [.1112, .3636] | 0.2352 | [.1088, .3615] |
| Non-Hispanic White (ref) |  |  |  |  |  |  |  |  |  |  |
| **Education** |  |  |  |  |  |  |  |  |  |  |
| Less than HS |  |  | 0.3567 | [.2661, .4473] | 0.2664 | [.1719, .3608] | 0.2167 | [.1217, .3117] | 0.2172 | [.1222, .3122] |
| HS Grad |  |  | 0.1245 | [.0645, .1844] | 0.1167 | [.0575, .1758] | 0.0902 | [.0309, .1494] | 0.0919 | [.0328, .151] |
| Some College |  |  | 0.084 | [-.0203, .1883] | 0.0807 | [-.0221, .1834] | 0.0617 | [-.0416, .1651] | 0.0637 | [-.0395, .1669] |
| College Grad and Above (ref) |  |  |  |  |  |  |  |  |  |  |
| **Household Income to Poverty Ratio** |  |  | -0.004 | [-.0065, -.0011] | -0.003 | [-.0054, -.0001] | -0.002 | [-.0045, .0008] | -0.002 | [-.0045, .0008] |
|  |  |  |  |  |  |  |  |  |  |  |
| ***Health Behaviors*** |  |  |  |  |  |  |  |  |  |  |
| **Smoking Status** |  |  |  |  |  |  |  |  |  |  |
| Current Smoker |  |  |  |  |  |  | 0.2534 | [.1567, .3502] | 0.2532 | [.1564, .3499] |
| Former Smoker |  |  |  |  |  |  | -0.006 | [-.0575, .0459] | -0.006 | [-.058, .0456] |
| Never Smoker (ref) |  |  |  |  |  |  |  |  |  |  |
| ***Health Status Indicators*** |  |  |  |  |  |  |  |  |  |  |
| **Change in Self-Reported Health** |  |  |  |  |  |  | 0.0001 | [-.0311, .0312] | 0.0001 | [-.0311, .0313] |
| **Self-Reported of a Change in Overall Health Status** |  |  |  |  |  |  |  |  |  |  |
| Somewhat better |  |  |  |  |  |  | 0.049 | [-.0286, .1267] | 0.049 | [-.0285, .1266] |
| Somewhat worse |  |  |  |  |  |  | -0.004 | [-.0662, .0593] | -0.004 | [-.0667, .0587] |
| Same (ref) |  |  |  |  |  |  |  |  |  |  |
| **Chronic Conditions Index** |  |  |  |  |  |  | 0.0165 | [-.0018, .0349] | 0.0165 | [-.0019, .0348] |
| **Change in Functional Limitations** |  |  |  |  |  |  | -0.007 | [-.0595, .0452] | -0.007 | [-.0595, .0449] |
| **n = 9,589** |  |  |  |  |  |  |  |  |  |  |

**Table S8.** Results of the regression analyses estimating the association between neighborhood affluence and the standardized CMV IgG.

|  | Model1: Neighborhood Affluence + Age + Gender | | Model2: Neighborhood Affluence + Age + Gender + SES | | Model3: Neighborhood Affluence + Age + Gender + SES + Race/Ethnicity | | Model4: Neighborhood Affluence + Age + Gender + SES + Race/Ethnicity + Health Behaviors and Chronic Conditions | | Model5: Neighborhood Affluence + Age + Gender + SES + Race/Ethnicity + Health Behaviors and Chronic Conditions + Neighborhood Density | |
| --- | --- | --- | --- | --- | --- | --- | --- | --- | --- | --- |
|  | β | 95% CI | β | 95% CI | β | 95% CI | β | 95% CI | β | 95% CI |
| **Neighborhood Characteristics** |  |  |  |  |  |  |  |  |  |  |
| Affluence | -0.01 | [-.0115, -.0083] | -0.007 | [-.009, -.0055] | -0.006 | [-.0073, -.0037] | -0.005 | [-.0069, -.0034] | -0.005 | [-.007, -.0035] |
| Density |  |  |  |  |  |  |  |  | 0 | [0, 0] |
|  |  |  |  |  |  |  |  |  |  |  |
| ***Demographics*** |  |  |  |  |  |  |  |  |  |  |
| **Age** (years) | 0.006 | [.0033, .0088] | 0.0042 | [.0015, .0069] | 0.0062 | [.0035, .009] | 0.0075 | [.0046, .0104] | 0.0075 | [.0045, .0104] |
| **Sex** (Female vs Male) | 0.2777 | [.2279, .3275] | 0.2683 | [.2187, .3179] | 0.2672 | [.2182, .3163] | 0.2725 | [.2221, .323] | 0.2721 | [.2217, .3225] |
| **Race/ Ethnicity** |  |  |  |  |  |  |  |  |  |  |
| Non-Hispanic Black |  |  |  |  | 0.3949 | [.3051, .4847] | 0.3714 | [.2805, .4623] | 0.3613 | [.2707, .4518] |
| Hispanic |  |  |  |  | 0.3181 | [.2284, .4078] | 0.3402 | [.2499, .4304] | 0.3243 | [.2331, .4156] |
| Other Race/Ethnicity |  |  |  |  | 0.2395 | [.1137, .3653] | 0.2506 | [.1247, .3766] | 0.245 | [.1189, .371] |
| Non-Hispanic White (ref) |  |  |  |  |  |  |  |  |  |  |
| **Education** |  |  |  |  |  |  |  |  |  |  |
| Less than HS |  |  | 0.3444 | [.2521, .4367] | 0.2269 | [.1303, .3234] | 0.1806 | [.0833, .2779] | 0.179 | [.0819, .2762] |
| HS Grad |  |  | 0.0892 | [.0269, .1515] | 0.081 | [.0199, .142] | 0.0572 | [-.004, .1184] | 0.0594 | [-.0017, .1204] |
| Some College |  |  | 0.0517 | [-.0529, .1563] | 0.0519 | [-.0508, .1545] | 0.035 | [-.0682, .1383] | 0.0383 | [-.0648, .1415] |
| College Grad and Above (ref) |  |  |  |  |  |  |  |  |  |  |
| **Household Income to Poverty Ratio** |  |  | -0.003 | [-.0059, -.0005] | -0.002 | [-.0045, .0008] | -0.001 | [-.0037, .0017] | -0.001 | [-.0036, .0017] |
|  |  |  |  |  |  |  |  |  |  |  |
| ***Health Behaviors*** |  |  |  |  |  |  |  |  |  |  |
| **Smoking Status** |  |  |  |  |  |  |  |  |  |  |
| Current Smoker |  |  |  |  |  |  | 0.2507 | [.1544, .347] | 0.2497 | [.1533, .346] |
| Former Smoker |  |  |  |  |  |  | -0.006 | [-.0572, .0463] | -0.006 | [-.058, .0455] |
| Never Smoker (ref) |  |  |  |  |  |  |  |  |  |  |
| ***Health Status Indicators*** |  |  |  |  |  |  |  |  |  |  |
| **Change in Self-Reported Health** |  |  |  |  |  |  | 0.0003 | [-.031, .0315] | 0.0004 | [-.0308, .0316] |
| **Self-Reported of a Change in Overall Health Status** |  |  |  |  |  |  |  |  |  |  |
| Somewhat better |  |  |  |  |  |  | 0.0484 | [-.0297, .1264] | 0.0482 | [-.0296, .1261] |
| Somewhat worse |  |  |  |  |  |  | -0.002 | [-.0647, .0602] | -0.004 | [-.0659, .0588] |
| Same (ref) |  |  |  |  |  |  |  |  |  |  |
| **Chronic Conditions Index** |  |  |  |  |  |  | 0.0147 | [-.0037, .0331] | 0.0143 | [-.0041, .0326] |
| **Change in Functional Limitations** |  |  |  |  |  |  | -0.007 | [-.0589, .0445] | -0.007 | [-.0589, .044] |
| **n = 9,589** |  |  |  |  |  |  |  |  |  |  |

**Table S9.** Results of the regression analyses evaluating the interaction between age, sex, and race/ethnicity and both neighborhood affluence and disadvantage across all four immune outcomes.

**Table S10.** Estimation of the average change in each of the immune measures with one-year of chronological age.

| Immune Measure | Change in the standard deviation associated with a 1-year higher value of chronological age |
| --- | --- |
| CD8:CD4 Ratio | 0.0122 (0.01, 0.014) |
| CD4, EMRA: Naïve | 0.0077 (0.0057, 0.0098) |
| CD8, EMRA: Naïve | 0.03 (0.027, 0.032) |
| CMV | 0.0078 (0.006, 0.010) |
